# Lymph node metastasis in young and middle-aged papillary thyroid carcinoma patients: a SEER-based cohort study

**DOI:** 10.1186/s12885-020-6675-0

**Published:** 2020-03-04

**Authors:** Yuanchao Liu, Yizeng Wang, Ke Zhao, Dongyang Li, Zuoyu Chen, Ruoyu Jiang, Xiaoning Wang, Xianghui He

**Affiliations:** Department of General Surgery, Tianjin Medical University General Hospital, 154 Anshan Road Heping District, Tianjin, 300052 China

**Keywords:** Papillary thyroid carcinoma, Age at diagnosis, Tumor size, Lymph node metastasis, SEER database

## Abstract

**Background:**

Lymph node metastasis (LNM) occurs frequently in young papillary thyroid carcinoma (PTC) patients, though the mortality rates are low. We aimed to analyze the relationship between age at diagnosis and LNM in PTC at a population level to elucidate the clinical behavior of PTC.

**Methods:**

Data of adult patients with surgically treated PTC and follicular thyroid carcinoma (FTC) were identified from the Surveillance, Epidemiology, and End Results (SEER) database (2004–2015) to investigate the relationship between age and clinical characteristics by curve estimation. The adjusted odds ratio of age and LNM rate were determined.

**Results:**

A total of 50,347 PTC (48,166) and FTC (2181) (median age: 45 and 50 years, respectively) patients met the inclusion criteria; 44.5% of those with PTC (21,428) had LNM. Rank-sum test analysis indicated differences in distribution of age in LNM-positive and LNM-negative PTC. The relationship between age, tumor size and LNM showed a quadratic curve in PTC. The mean tumor diameter and LNM rate correlated linearly with age in 18–59-year-old patients. LNM rate decreased with age (*R*^*2*^ = 0.932, *P* < .0001), especially women (*R*^*2*^ = 0.951, *P* < .0001).

**Conclusion:**

In young and middle-aged PTC patients, LNM may resolve spontaneously with delayed diagnosis and management. Active surveillance of low-risk PTC is justified.

## Background

In the United States (US), thyroid cancer is the most rapidly increasing cancer and more than 50,000 new cases will be diagnosed in 2019, with an increasing death rate [1, 2]. Differentiated thyroid carcinoma (DTC), appearing mainly as papillary thyroid carcinoma (PTC) accounts for approximately 90% of the disease [3]. By the year 2019, PTC is expected to become the third most common cancer in women in the US [4].

PTC occurs predominantly in young and middle-aged individuals, is less aggressive, and is associated with a low mortality rate. Patient age is a strong predictor of patient outcomes, and recurrence and death rates are significantly higher in older patients [5, 6]. Age at diagnosis is incorporated into all major thyroid cancer staging systems, including the American Joint Committee on Cancer (AJCC) staging system [7, 8]. Young and middle–aged PTC patients are classified into stages I and II, regardless of local extension and metastasis.

Active surveillance in papillary thyroid microcarcinoma (PTMC) is recommended but not well accepted because of the concern of lymph node metastasis (LNM). Previously, LNM was recognized as a prognostic indicator only in older PTC and follicular thyroid carcinoma (FTC) patients [9]. Recently, Adam et al. [10] demonstrated that in young PTC patients, the presence of cervical LNM and the number of metastatic cervical lymph nodes are associated with compromised survival. LNM predicts poor prognosis and decreased overall survival (OS) in PTC. However, LNM incidence is high while mortality is low in young patients. Therefore, the relationship among age at diagnosis, LNM, and OS is still inconclusive. It is difficult to monitor the fate of metastatic lymph nodes because lymph node status cannot be ascertained without surgery. The aim of this study was to analyze the relationship between the age at diagnosis and LNM in PTC at the population level, to elucidate the clinical behavior of PTC.

## Methods

### SEER database

We extracted data from the Surveillance, Epidemiology, and End Results (SEER) cancer registry maintained by the National Cancer Institute, which currently collects and publishes cancer incidence, survival data, therapy information, and clinicopathological characteristics of patients from population-based cancer registries covering approximately 34.6% of the US population.

### Patient selection

We identified patients diagnosed with PTC (ICD-O-3 codes 8050, 8260–8263, 8340–8344, 8450) and FTC (ICD-O-3 codes 8290, 8330–8333, 8335) between 2004 and 2015. Variables included patient age at diagnosis, sex, race, tumor size, extrathyroidal extension (ETE), laterality, multifocality, histological variant, LNM, and distant metastasis at initial treatment.

Exclusion criteria included age < 18 years, presence of other primary cancers, lack of pathological confirmation of cancer, did not undergo lymphadenectomy, and unknown LNM status. Patients with unknown race, tumor size, ETE, multifocality, histological variant, and distant metastasis status were excluded from multivariate logistic regression analysis.

### Statistical analysis

Descriptive statistics were used to analyze patient characteristics by LNM status; continuous variables were compared using Wilcoxon rank sum tests, and categorical variables by Pearson χ^2^ or the exact Fisher’s tests. The distribution of age at diagnosis was compared by Wilcoxon rank sum tests or Kruskal-Wallis H tests according to demographic and clinicopathological characteristics. The odds ratio (OR) for relationships between clinicopathological factors and LNM were calculated using multivariate logistic regression.

We further investigated the relationship of age at diagnosis with tumor size, LNM, ETE, and distant metastasis by Spearman rank correlation and curve estimation.

To analyze the trend in rate change in tumor size and LNM by age in adults with normal immunologic function, we selected PTC patients aged 18 to 59 years and classified them by sex and T stage (as per AJCC, 8th edition) [11].

To systematically analyze the contribution of age toward tumor size and LNM in PTC, multivariate analyses were performed, with age (18 to 59 years) as a categorical variable. Furthermore, to evaluate the change in protective effect of age, the age cutoffs were incrementally stepped from 21 to 57 years in 1-yearly increments, and the adjusted OR for older age was compared across multivariate logistic regression models. The effects of the following were adjusted for in the model: sex, race, tumor size, ETE, multifocality, histology, and distant metastases.

Two-sided *P*-values <.05 were considered significant. Statistical analyses were performed using SPSS Statistics version 22 (IBM Corp., Armonk, NY, USA) and GraphPad Prism 7 (GraphPad Software, La Jolla, California, USA).

### Ethical approval

We received permission to access the research data file in the SEER program from the National Cancer Institute, US (reference number 15218-Nov2017). Approval was waived by the local ethics committee, as SEER data is publicly available and de-identified.

## Results

### Demographic and clinical characteristics of DTC according to LNM status

A total of 50,347 surgically treated PTC (48,166) and FTC (2181) patients met the inclusion criteria (women, 77.4% and White race, 82.9%); 44.5% of PTC (21,428) had LNM. The median age was 45 years (range, 18–99 years) for PTC and 50 years (18–94) for FTC. The mean tumor size was 19.3 ± 17.6 mm for 48,166 PTC; 15,721 (32.6%) of PTC were small (PTMC; maximum diameter, ≤1 cm). Table 1 presents the patient demographic and clinicopathological characteristics. For PTC, the mean age at diagnosis was lower in the LNM-positive group compared to that in the LNM-negative group (43.8 ± 14.7 vs. 47.4 ± 13.6 years, respectively; *P* < .0001) (Fig. S1). LNM-positive and LNM-negative PTC patients differed in the following parameters: sex, tumor size, ETE, multifocality, histological variant, T stage, and distant metastasis (Table 2). PTC patients with male sex, Black race, small tumor size, major neck structure invasion, multifocality, columnar cell variant, T1a and T4 stage, LNM, and distant metastasis were more frequently diagnosed at an older age (Rank sum test analysis, Table 2).

**Table 1:** Demographic and Clinicopathological Characteristics of Thyroid Carcinoma Patients according to Lymph Node Metastasis Status

Variance	PTC				FTC			
	LNM-negative	LNM-positive	Total	***P*** value	LNM-negative	LNM-positive	Total	***P*** value
**Age at diagnosis (mean ± SD),y**	47.4 ± 13.6	43.8 ± 14.7	45.8 ± 14.2	**<.0001**	48.8 ± 15.5	55.3 ± 16.3	49.6 ± 15.7	**<.0001**
**Sex**				**<.0001**				**<.0001**
Male	4349 (40.3)	6443 (59.7)	10,792		470 (81.3)	108 (18.7)	578	
Female	22,389 (59.9)	14,985 (40.1)	37,374		1450 (90.5)	153 (9.5)	1603	
**Race**				**<.0001**				**.004**
White	22,468 (56.3)	17,472 (43.7)	39,940		1599 (88.7)	203 (11.3)	1802	
Black	1166 (64.1)	654 (35.9)	1820		136 (88.3)	18 (11.7)	154	
API	2613 (48.2)	2812 (51.8)	5425		0	0	0	
AI/AN	119 (38.6)	189 (61.4)	308		157 (80.1)	39 (19.9)	196	
Unknown	372 (55.3)	301 (44.7)	673		28 (96.6)	1 (3.4)	29	
**Tumor size (mean ± SD),mm**	16.1 ± 15.2	23.3 ± 19.5	19.3 ± 17.6	**<.0001**	34.7 ± 23.0	43.9 ± 29.0	35.8 ± 23.9	**<.0001**
0–10	11,448 (72.8)	4273 (27.2)	15,721		146 (89.6)	17 (10.4)	163	
11–20	8195 (52.8)	7336 (47.2)	15,531		352 (89.3)	42 (10.7)	394	
21–40	5168 (43.7)	6668 (56.3)	11,836		808 (91.2)	78 (8.8)	886	
>40	1507 (37.0)	2568 (63.0)	4075		562 (83.1)	114 (16.9)	676	
Unknown	420 (41.9)	583 (58.1)	1003		52 (83.9)	10 (16.1)	62	
**ETE**				**<.0001**				**<.0001**
Localized	23,577 (65.2)	12,586 (34.8)	36,163		1693 (93.0)	128 (7.0)	1821	
Minor^a^	1091 (26.4)	3036 (73.6)	4127		59 (61.5)	37 (38.5)	96	
Strap muscles^b^	1453 (30.3)	3341 (69.7)	4794		68 (71.6)	27 (28.4)	95	
Major neck structures^c^	373 (16.8)	1841 (83.2)	2214		63 (53.4)	55 (46.6)	118	
Unknown	244 (28.1)	624 (71.9)	868		37 (72.5)	14 (27.5)	51	
**Laterality**				**.03**				.55
One side	26,584 (55.6)	21,270 (44.4)	47,854		1914 (88.1)	259 (11.9)	2173	
Bilateral	154 (49.4)	158 (50.6)	312		6 (75.0)	2 (25.0)	8	
**Multifocality**				**<.0001**				**<.0001**
Absent	15,054 (62.4)	9079 (37.6)	24,133		1587 (91.4)	149 (8.6)	1736	
Present	11,387 (49.1)	11,814 (50.9)	23,201		298 (76.2)	93 (23.8)	391	
Unknown	297 (35.7)	535 (64.3)	832		35 (64.8)	19 (35.2)	54	
**Histological Variant**				**<.0001**				
Adenocarcinoma	14,709 (47.8)	16,065 (52.2)	30,774		–	–	–	
Follicular variant	9168 (71.5)	3650 (28.5)	12,818		–	–	–	
Oxyphilic cell variant	52 (56.5)	40 (43.5)	92		–	–	–	
Encapsulated variant	209 (76.0)	66 (24.0)	275		–	–	–	
Columnar cell variant	267 (35.6)	484 (64.4)	751		–	–	–	
Microcarcinoma^d^	1412 (85.3)	244 (14.7)	1656		–	–	–	
Unknown	921 (51.2)	879 (48.8)	1800		–	–	–	
**T Stage**				**<.0001**				**<.0001**
T1a	11,146 (75.3)	3652 (24.7)	14,798		144 (90.6)	15 (9.4)	159	
T1b	7497 (56.5)	5763 (43.5)	13,260		342 (91.2)	33 (8.8)	375	
T2	4641 (49.2)	4801 (50.8)	9442		773 (93.1)	57 (6.9)	830	
T3	2746 (36.2)	4850 (63.8)	7596		551 (85.6)	93 (14.4)	644	
T4	373 (16.8)	1842 (83.2)	2215		63 (53.4)	55 (46.6)	118	
Unknown	335 (39.2)	520 (60.8)	855		47 (85.5)	8 (14.5)	55	
**Distant metastasis**^**e**^				**<.0001**				**<.0001**
No	26,497 (56.1)	20,698 (43.9)	47,195		1856 (90.1)	205 (9.9)	2061	
Yes	103 (18.5)	455 (81.5)	558		56 (50.9)	54 (49.1)	110	
Unknown	138 (33.4)	275 (66.6)	413		8 (80.0)	2 (20.0)	10	
**Total**	26,738 (55.5)	21,428 (44.5)	48,166		1920 (88.0)	261 (12.0)	2181	

**Abbreviations**: *LNM* Lymph node metastasis, *PTC* Papillary thyroid carcinoma *FTC* Follicular thyroid carcinoma, *API* Asian or Pacific Islander, *AI/AN* American Indian/Alaska Native, *ETE* Extrathyroidal extension

**Note**: ^a^Pericapsular soft tissue/connective tissue; ^b^sternohyoid, sternothyroid, thyrohyoid, or omohyoid muscles; ^c^invading subcutaneous soft tissues, larynx, trachea, esophagus, recurrent laryngeal nerve, prevertebral fascia, encasing carotid artery, or mediastinal vessels; ^d^labeled as microcarcinoma in the Surveillance Epidemiology, and End Results (SEER) database, there was no specific pathological subtype classification; ^e^including the bone, brain, liver, and lung. Number of observations: 50,347

**Table 2:** The Distribution of Age at Diagnosis in Thyroid Carcinoma Patients according to Demographic and Clinicopathological Characteristics

Variance	PTC			FTC		
	Mean ± SD, year	Median (QR), year	***P*** value^**a**^	Mean ± SD, year	Median (QR), year	***P*** value^**a**^
**Sex**			**<.0001**			**<.0001**
Male	48.6 ± 14.3	48 (20)		53.1 ± 14.5	54 (19)	
Female	45.0 ± 14.1	44 (20)		48.3 ± 16.0	48 (23)	
**Race**			**<.0001**			.12
White	45.8 ± 14.3	45 (20)		49.6 ± 15.7	50 (23)	
Black	46.6 ± 13.7	45 (19)		49.9 ± 15.0	50 (22)	
API	45.7 ± 14.1	45 (21)		–	–	
AI/AN	42.6 ± 14.5	40 (22)		49.9 ± 16.2	49 (24)	
Unknown	42.1 ± 13.3	41 (19)		42.5 ± 14.1	40 (23)	
**Tumor size (mean ± SD),mm**			**<.0001**			**<.0001**
0–10	47.3 ± 13.3	47 (19)		48.6 ± 13.9	50 (19)	
11–20	45.1 ± 13.7	44 (20)		47.9 ± 14.9	47 (21)	
21–40	44.5 ± 15.0	43 (22)		47.9 ± 15.1	48 (23)	
>40	46.0 ± 16.3	44 (24)		52.9 ± 16.7	53 (26)	
Unknown	47 ± 15.2	46 (22)		51.5 ± 16.6	53 (27)	
**ETE**			**<.0001**			**<.0001**
Localized	45.1 ± 13.8	44 (20)		47.9 ± 15.1	48 (23)	
Minor^b^	46.3 ± 14.7	46 (21)		57.8 ± 16.4	57.5 (23)	
Strap muscles^c^	46.5 ± 14.7	46 (22)		53.5 ± 16.8	55 (26)	
Major neck structures^d^	53.2 ± 16.7	53 (26)		61.3 ± 14.3	63 (17)	
Unknown	47.1 ± 15.1	47 (23)		60.0 ± 15.3	59 (24)	
**Laterality**			.49			.72
One side	45.8 ± 14.2	45 (20)		49.6 ± 15.7	50 (23)	
Bilateral	46.4 ± 14.5	45 (21)		52.5 ± 19.5	48.5 (33)	
**Multifocality**			**.01**			**<.0001**
Absent	45.6 ± 14.5	45 (21)		49.1 ± 15.7	49 (23)	
Present	45.9 ± 13.9	45 (20)		50.9 ± 15.4	51 (21)	
Unknown	46.4 ± 15.1	45.5 (21)		57.5 ± 15.9	57 (24)	
**Histological Variant**			**<.0001**			–
Adenocarcinoma	44.9 ± 14.3	44 (21)		–	–	
Follicular variant	47.3 ± 13.9	47 (20)		–	–	
Oxyphilic cell variant	47.6 ± 15.5	48.5 (23)		–	–	
Encapsulated variant	46.0 ± 12.8	45 (19)		–	–	
Columnar cell variant	50.5 ± 15.5	50 (23)		–	–	
Microcarcinoma^e^	47.7 ± 13.0	48 (19)		–	–	
Unknown	45.0 ± 14.2	44 (20)		–	–	
**T Stage**			**<.0001**			**<.0001**
T1a	47.2 ± 13.2	47 (19)		48.8 ± 13.9	50 (18)	
T1b	44.7 ± 13.6	44 (19)		47.5 ± 15.0	47 (21)	
T2	43.5 ± 14.5	42 (21)		47.3 ± 14.9	47 (22)	
T3	45.6 ± 15.0	45 (22)		51.7 ± 16.6	52 (26)	
T4	53.2 ± 16.7	53 (26)		61.3 ± 14.3	63 (17)	
Unknown	45.6 ± 14.6	45 (21)		50.4 ± 17.0	51 (27)	
**Lymph node metastasis**			**<.0001**			**<.0001**
Negative	47.4 ± 13.6	47 (20)		48.8 ± 15.5	49 (23)	
Positive	43.8 ± 14.7	42 (22)		55.3 ± 16.3	56 (24)	
**Distant metastasis**^**f**^			**<.0001**			**<.0001**
No	45.7 ± 14.1	45 (20)		48.8 ± 15.4	49 (23)	
Yes	55.6 ± 17.2	58 (24)		64.9 ± 13.0	67 (17)	
Unknown	43.9 ± 14.9	43 (22)		49.7 ± 16.9	48.5 (31)	
**Total**	45.8 ± 14.2	45 (20)		49.6 ± 15.7	50 (23)	

**Abbreviations**: *PTC* Papillary thyroid carcinoma, *FTC* Follicular thyroid carcinoma, *SD* Standard deviation, *QR* Quartile, *API* Asian or Pacific Islander, *AI/AN* American Indian/Alaska Native, *ETE* Extrathyroidal extension

**Note**: ^a^*P* value was calculated by Wilcoxon rank sum tests or by Kruskal-Wallis H test; ^b^Pericapsular soft tissue/connective tissue; ^c^sternohyoid, sternothyroid, thyrohyoid, or omohyoid muscles; ^d^ invading subcutaneous soft tissues, larynx, trachea, esophagus, recurrent laryngeal nerve, prevertebral fascia, or encasing the carotid artery or mediastinal vessels; ^e^labeled as microcarcinoma in Surveillance, Epidemiology, and End Results (SEER) database, there was no specific pathological subtype classification; ^f^including the bone, brain, liver, and lung. Number of observations: 50,347

### Risk factors for LNM

There was adequate information for 43,344 PTC and 2002 FTC patients to be included in the multivariate analysis. Multivariate logistic regression showed that male sex, large tumor size, ETE, multifocality, and distant metastasis significantly increased LNM risk in PTC (Table 3). In FTC, LNM risk was significantly increased with male sex, ETE, multifocality, and distant metastasis (Table 3). Importantly, age at diagnosis decreased the risk of LNM in PTC, with OR (95% confidence interval (CI)) 0.974 (0.972–0.975) (*P* < .0001). In contrast, for FTC, age at diagnosis was not related to the risk of LNM.

**Table 3:** Multivariate Logistic Regression Analysis of Demographic and Clinicopathological Characteristics Predictive of Lymph Node Metastasis in Thyroid Carcinoma Patients

Variance	PTC		FTC	
	OR(95%CI)	***P*** value	OR(95%CI)	***P*** value
**Age at diagnosis**	0.974 (0.972–0.975)	**<.0001**	0.999 (0.988–1.009)	.82
**Sex (female vs. male)**	0.481 (0.456–0.507)	**<.0001**	0.569 (0.408–0.796)	**.001**
**Race**		**<.0001**		**.02**
White	1 (reference)		1 (reference)	
Black	0.782 (0.695–0.881)	**<.0001**	1.010 (0.530–1.923)	.98
API	1.191 (1.114–1.274)	**<.0001**	–	–
AI/AN	1.714 (1.300–2.261)	**<.0001**	1.883 (1.193–2.971)	**.007**
**Tumor size (mm)**		**<.0001**		.51
0–10	1 (reference)		1 (reference)	
11–20	1.821 (1.724–1.923)	**<.0001**	1.291 (0.629–2.652)	.49
21–40	2.36 (2.224–2.504)	**<.0001**	0.934 (0.473–1.843)	.84
>40	2.547 (2.329–2.784)	**<.0001**	0.952 (0.472–1.919)	.89
**ETE**		**<.0001**		**<.0001**
Localized	1 (reference)		1 (reference)	
Minor^a^	4.127 (3.805–4.478)	**<.0001**	6.627 (3.974–11.050)	**<.0001**
Strap muscles^b^	3.509 (3.260–3.777)	**<.0001**	4.497 (2.621–7.715)	**<.0001**
Major neck structures^c^	7.575 (6.641–8.640)	**<.0001**	7.620 (4.580–12.676)	**<.0001**
**Laterality**	0.950 (0.714–1.265)	.73	–	1.00
**Multifocality**	1.661 (1.590–1.735)	**<.0001**	2.816 (2.012–3.941)	**<.0001**
**Histology**		**<.0001**		–
Adenocarcinoma	1 (reference)		–	
Follicular variant	0.371 (0.353–0.391)	**<.0001**	–	–
Oxyphilic cell variant	0.741 (0.470–1.168)	.20	–	–
Encapsulated variant	0.312 (0.230–0.424)	**<.0001**	–	–
Columnar cell variant	1.089 (0.913–1.298)	.34	–	–
Microcarcinoma^d^	0.338 (0.290–0.393)	**<.0001**	–	–
**Distant metastasis**^**e**^	2.787 (2.137–3.635)	**<.0001**	3.768 (2.195–6.467)	**<.0001**

**Abbreviations**: *PTC* Papillary thyroid carcinoma, *FTC* Follicular thyroid carcinoma, *OR* Odds ratio, *CI* Confidence interval, *API* Asian or Pacific Islander, *AI/AN* American Indian/Alaska Native, *ETE* Extrathyroidal extension

**Note**: ^a^Pericapsular soft tissue/connective tissue; ^b^sternohyoid, sternothyroid, thyrohyoid, or omohyoid muscles; ^c^invading subcutaneous soft tissues, larynx, trachea, esophagus, recurrent laryngeal nerve, prevertebral fascia, or encasing the carotid artery or mediastinal vessels; ^d^labeled as microcarcinoma in Surveillance, Epidemiology, and End Results (SEER) database, there was no specific pathological subtype classification; ^e^including the bone, brain, liver, lung. **Reference groups** are male, white, tumor size≤10 mm, intrathyroidal tumors, one side tumors, single tumors, adenocarcinoma, and absence of distant metastasis. Number of observations: 45,346. Bolded font indicates statistical significance

### Correlation analysis of age at diagnosis and tumor size, LNM, ETE, and distant metastasis in PTC patients

**Fig. 1:**
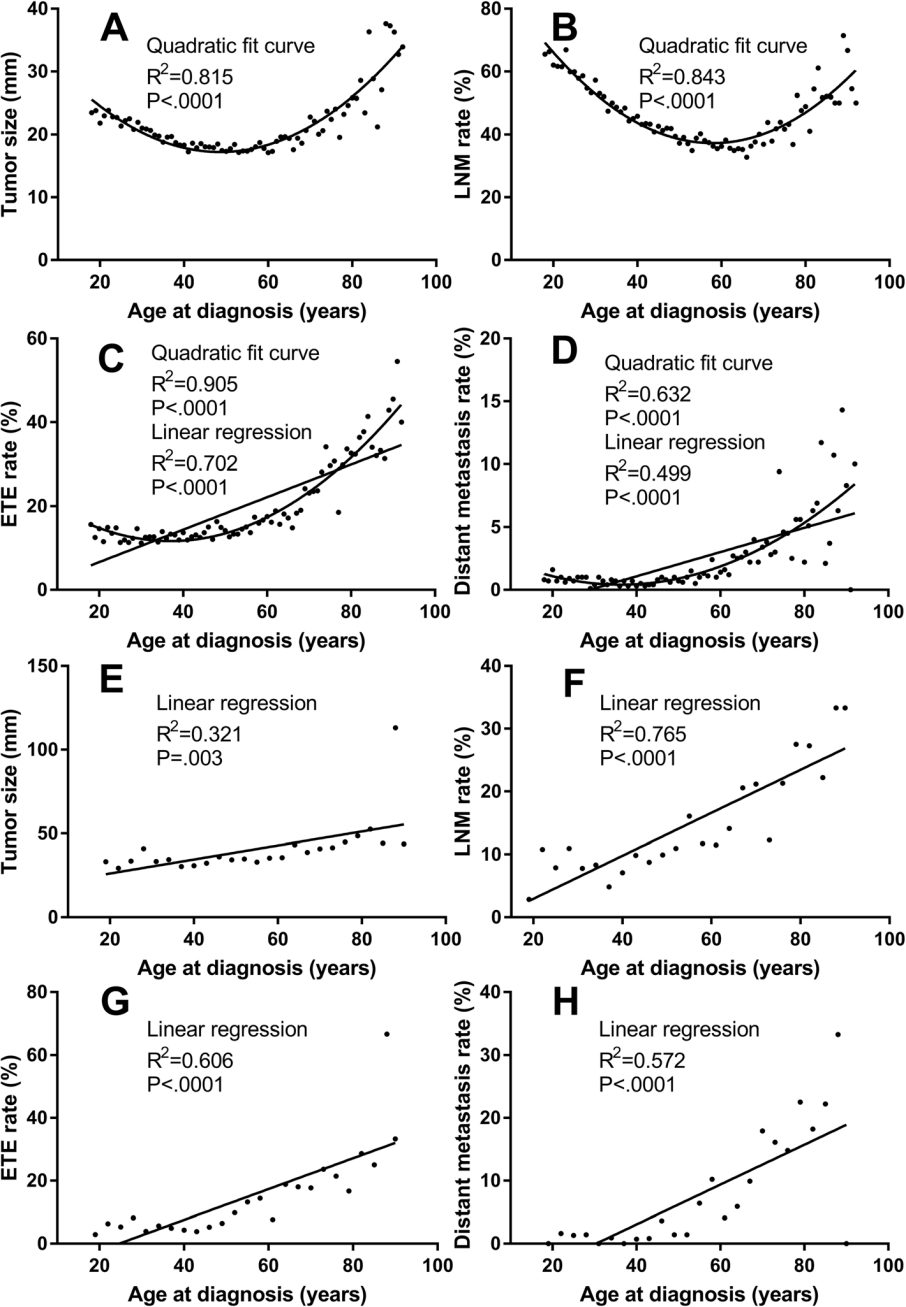
Curve-fitting analysis of age at diagnosis and tumor size, lymph node metastasis (LNM), extrathyroidal extension (ETE), and distant metastasis in papillary thyroid carcinoma (PTC) and follicular thyroid carcinoma (FTC) patients. **Abbreviations**: ETE, extrathyroidal extension. Tumor size was represented by the mean tumor diameter; and LNM, ETE, and distant metastasis were expressed as positivity rates. Number of observations: **a**, 46,884; **b**, 48,166; **c**, 47,298; **d**, 47,753; **e**, 2107; **f**, 2181; **g**, 2130; **h**, 2171

We first did Spearman rank correlation analysis and results indicated that there was correlation between tumor size, LNM, ETE, distant metastasis and age at diagnosis (treated as a continuous variable, 18 to > 92 years). Tumor size was expressed as the mean tumor diameter, and LNM, ETE, and distant metastasis as positive rates. Spearman correlation coefficients (r_s_) yielded was 0.307 (*P* = .007), − 0.266 (*P* = .02), 0.877 (*P* < .0001), and 0.751 (*P* < .0001) respectively in PTC. To further explore the correlation between tumor size, LNM, ETE, distant metastasis and age at diagnosis. Curve estimation and regression analysis were performed. As shown in Fig. 1a-d, the relationship between tumor size and age at diagnosis fit nicely with a quadratic function (*R*^*2*^ = 0.815); Similar relationships were shown between LNM, ETE and age at diagnosis, (*R*^*2*^ = 0.843 and *R*^*2*^ = 0.905 respectively). It is worth noting that the bottom of the curve corresponding to patients at middle age (40–60 years old). The left portion of the curve indicated that the mean tumor diameter and LNM rate decreased with age in young and middle-aged PTC patients.

### Correlation analysis of age at diagnosis and tumor size, LNM, ETE, and distant metastasis in FTC patients

Although multivariate analysis of FTC patients showed no correlation between age at diagnosis and LNM, to compare with PTC patients, we also performed Spearman rank correlation and curve estimation of tumor size, LNM, ETE, distant metastasis, and age at diagnosis (treated as a continuous variable, 18 to > 90 years) in FTC patients. Because the number of FTC patients was small and the Y value contained several 0 values, we used 3-yearly increments for analysis. Spearman correlation coefficient (r_s_) for tumor size (mean tumor diameter), LNM, ETE, and distant metastasis (positive rate) were 0.820 (*P* < .0001), 0.909 (*P* < .0001), 0.890 (*P* < 0.0001), and 0.700 (*P* < .0001), respectively. Curve estimation analysis results including the best match model and regression coefficients are shown in Fig. 1e-h. The relationship between tumor size, LNM, ETE, distant metastasis and age at diagnosis for FTC were totally different from that of the PTC. Further analysis of age at diagnosis and LNM was focused on PTC.

### Relationship between tumor size and LNM rate, and age at diagnosis in young and middle-aged PTC patients

**Fig. 2:**
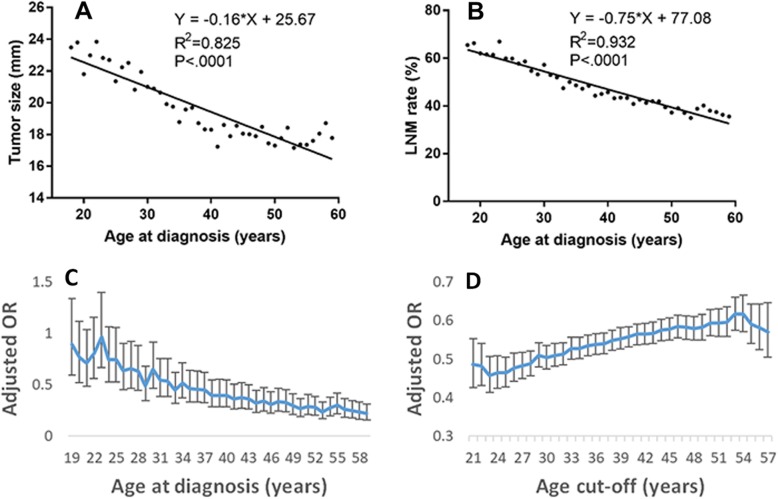
**a, b** Correlation analysis between age at diagnosis and tumor size or lymph node metastasis (LNM) in patients with papillary thyroid carcinoma (PTC) aged from 18 to 59 years. Age at diagnosis correlated linearly with tumor size and LNM; tumor size and LNM rate decreased with age (*R*^*2*^ = 0.825, *P* < .0001 for tumor size and 0.932, *P* < .0001 for LNM). Tumor size was represented by the mean tumor diameter; LNM was expressed as positive rate. **c.** Adjusted odds ratio (OR) between patient age at diagnosis and LNM in patients with PTC aged from 18 to 59 years. Covariates included age, sex, race, tumor size, extrathyroidal extension (ETE), multifocality, histology, and distant metastases. Adjusted OR was calculated with reference to 18-year-old PTC patients. The vertical whisker bars represent the 95% confidence intervals (CI). **d.** Adjusted OR between patient age cut-off and LNM in PTC patients aged from 18 to 59 years. Covariates included: sex, race, tumor size, ETE, multifocality, histology, and distant metastases. Age cut-off was also a covariate, and was incrementally stepped from 21 to 57 years in 1-year increments. The vertical axis represents the adjusted OR for the older group, compared to that of the younger group. The vertical whisker bars represent the 95% confidence intervals (CI). Number of observations: A, 38,741; B, 39,751; C and D, 35,753

Statistical analysis showed that the relationship between age at diagnosis and tumor size and LNM fit a quadratic curve in all age groups of PTC patients. Biologically, young and middle-aged patients usually have normal immune function which may affect tumor growth and metastasis. We further selected patients with PTC from those aged 18–59 years to analyze the correlation between age at diagnosis and tumor size, and between age at diagnosis and LNM**.** Age at diagnosis correlated linearly with tumor size and LNM; tumor size and LNM rate decreased with age ( 0.825, *P* < .0001 for tumor size; 0.932, *P* < .0001 for LNM) (Fig. 2a, b).

To analyze the contribution of age towards LNM in PTC, multivariate analyses were performed with age, from 18 to 59 years, as a categorical variable. The adjusted OR was calculated with reference to the 18-year-old PTC patients. The adjusted OR value was < 1, which implied that age was a protective factor for LNM in comparison with the 18-year-old PTC patients (Fig. 2c).

To analyze changes in protective effects of age; the age cutoffs were incrementally stepped from 21 to 57 years in 1-yearly increments, and the adjusted OR for older age was compared across multivariate logistic regression models. The adjusted ORs for PTC patients aged 18–59 years are plotted in Fig. 2d. The adjusted OR for advanced age cut-off in PTC increased slightly and (statistically significant, *P* < .0001) in all models, implying that the protective effect of age on LNM gradually weakened with increasing age.

### Correlation analysis of age at diagnosis and LNM rate in PTC patients aged 18–59 years

**Fig. 3:**
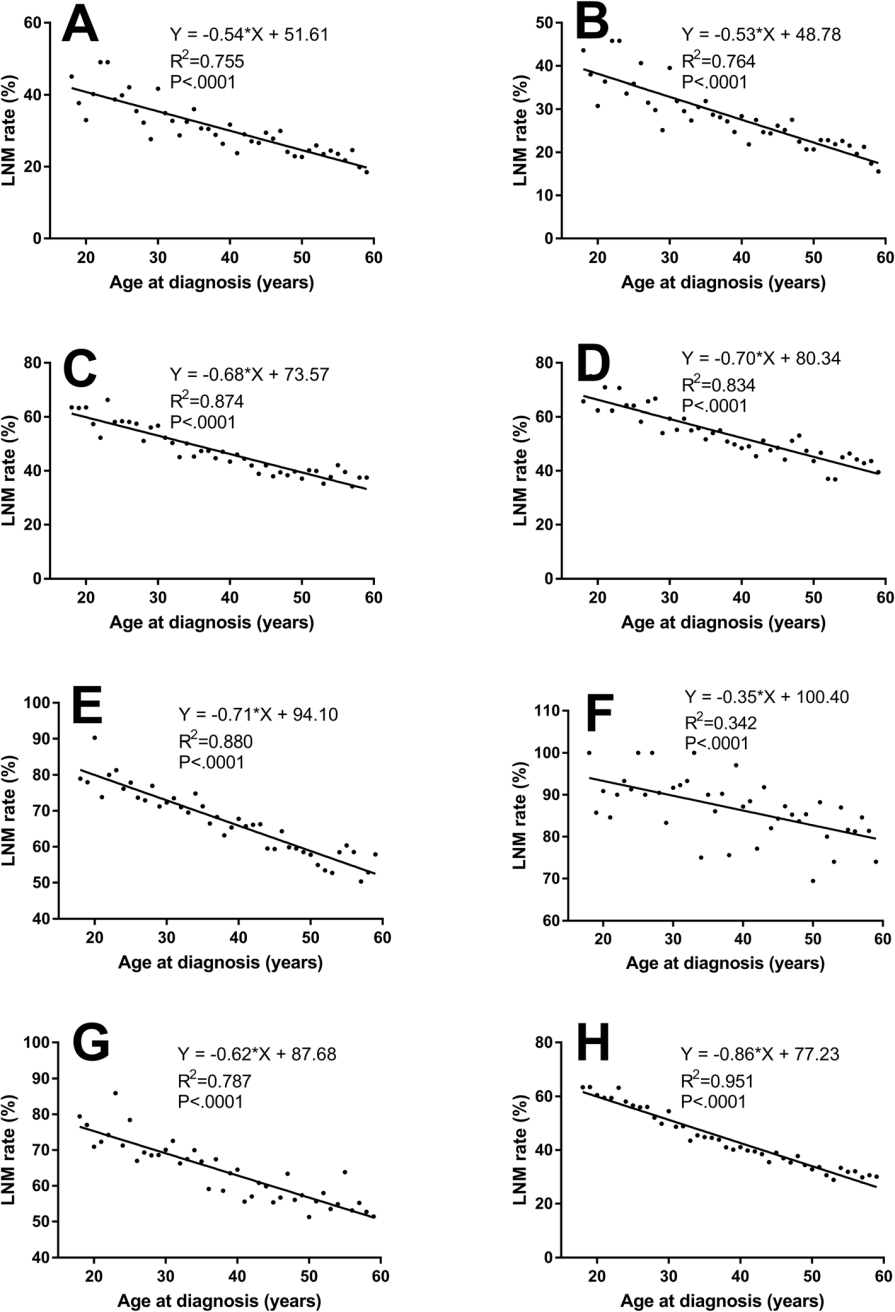
Correlation analysis of age at diagnosis and LNM rate in patients with papillary thyroid carcinoma (PTC) aged from 18 to 59, and classified by T stage and sex; **a.** Total papillary thyroid microcarcinoma (PTMC) patients, *N* = 12,796; **b.** T0 and T1a PTC patients, *N* = 12,056; **c.** T1b PTC patients, *N* = 11,339; **d.** T2 PTC patients, *N* = 8044; **e.** T3 PTC patients, *N* = 6197; **f.** T4 PTC patients, *N* = 1397; **g.** Male PTC patients, *N* = 8341; **h.** Female PTC patients, *N* = 31,410

To analyze the correlation between age at diagnosis and LNM rate in adults with normal immunologic function, we selected PTC patients aged 18–59 years and also classified them by T stage and sex. As shown in Fig. 3 and Table S1, in all T stages, LNM rates were associated inversely with age at diagnosis. The same trend was observed for sex; the LNM rate in women (*R*^*2*^ = 0.951, *P* < .0001) decreased more rapidly than it did in men.

## Discussion

We analyzed demographic and clinicopathological characteristics of patients with PTC and FTC, and showed that younger PTC patients had larger tumor sizes and higher LNM rates (rank-sum tests). We identified that LNM rate decreased with age in young and middle-aged adult PTC patients, implying that younger PTC patients had an increased predisposition for LNM, regardless of T stage or sex.

In clinical practice, it is unjustified to follow-up a patient with preoperative diagnosis of PTC with LNM without surgery; thus, it is difficult to directly assess the unperturbed natural history of a given lesion [12]. Additionally, although radioguided occult localization technique could be used to localize the lesion site of cervical recurrences from DTC in a study [13], LNM status can still not be accurately assessed before surgery in clinical work. Here, we used population data to evaluate changes in LNM incidence with age in PTC patients aged 18–59 years. Results in this study showed that LNM rate decreases with age in PTC patients. We speculate that LNM may disappear spontaneously and tumors may shrink in size in some young and middle-aged PTC patients with delayed management. Because young and middle-aged individuals have normal immunologic responses, the activation of anti-tumor immunity could be the underlying mechanism resulting in tumor shrinkage and spontaneous resolution of LNM. Furthermore, the activation of the immune system leads to a lower risk and better survival of PTC patients.

Earlier reports of the active surveillance of PTMC cases revealed that in each follow-up period, 4.2–16.6% of the PTMC decreased in size compared to the size at initial observation [14]. In 2010, the Cancer Institute Hospital in Tokyo, Japan reported that of 300 PTMC lesions, 3% had decreased in tumor size during active surveillance [15]. Recent data show that the proportions of tumors which decreased in size was 17%, with a distribution of 15, 21, and 13% for age at presentation of ≤40, 41–60, and > 60 years, respectively [16]. A retrospective observational study from Italy [17] showed that 34.9% (15/43) of indeterminate and 3.8% (2/52) of suspicious lymph nodes had disappeared during follow-up. These longitudinal data from cohorts of patients who do not undergo surgery or after thyroidectomy surgery supported that LNM may resolve spontaneously and tumors may shrink in some young and middle-aged PTC patients.

A number of studies [18–22] have examined the association between patient age at diagnosis and LNM in PTC, but the conclusions have been inconsistent. A single institution study of 1226 patients with PTMC [18] demonstrated that middle-aged and elderly patients had a lower risk of LNM compared with young patients. Similar to our study, Andrew et al. [20] found that the ipsilateral central LNM risk related to age was U-shaped with increased odds of metastases in younger and older patients. The number of cases analyzed in these studies was not large enough to derive confirmative results. A recent SEER study examined the association between patient age and LNM in 46,077 patients with PTC (1998 to 2013), [22] and showed that younger patients had an increased LNM risk regardless of T stage. However, since patients were grouped based on 10–yearly age increments, it was impossible for them to infer that LNM can disappear spontaneously with delayed surgery in immune competent young and middle-age patients.

The paradox that young PTC patients showed a higher LNM rate but a lower mortality highlights the need for further investigation into age-related differences in PTC biology, lymph node immunologic function, immunological surveillance, and host response. Previous data showed that positive lymph nodes were immune-activated in PTC patients. According French et al. [23], Th1 polarization and high levels of interferon (IFN)-γ^+^/CD8^+^ T cells were observed in many tumor-involved lymph nodes in primary and recurrent PTC. Thus, they believed that the presence of metastases did not deter, and may have promoted, an IFN-γ response. Proliferating lymphocytes and Th1-polarization in many tumor-involved lymph nodes suggest that a productive antitumor response had been generated, which may impair tumor evasion [23]. Another study performed a functional analysis of tumor-associated lymph nodes and showed that CD8^+^ T-cell exhaustion is incomplete and may be reversible in DTC patients, and that proliferative capacity can be largely maintained in DTC-associated T cells [24]. In addition, the good response to radioactive iodine also played a role in support of the higher LNM rate but with lower mortality rate in younger PTC patients.

The trend to an inverse relationship between incidence of LNM and patient age at diagnosis was also demonstrated in melanoma, and lung, rectum, and breast cancer [25–29]. Data from thyroid cancer, early-stage rectal cancer, and breast cancer imply that LNM of some low-risk cancers may spontaneously disappear in immunocompetent young and middle-aged patients.

The SEER database is a powerful tool for use in clinical research, since it contains detailed information on tumor characteristics, demographic data, survival status, surgical interventions, and radiation therapy [30]. Our study included detailed demographic and clinicopathological characteristics of a cohort of 48,166 PTC and 2181 FTC patients; the data were adequately large and allowed us to perform multivariable analysis after accounting for confounders. However, there are several limitations to the current study. Since SEER only captured first line treatment data and survival status, subsequent treatment and recurrence data after surgery were not included. Our study did not include follow-up analyses, and only LNM at initial surgery was included. Data on thyroiditis, hyperthyroidism, and other thyroid diseases was lacking, so the relationship between LNM, age, and thyroid autoimmune diseases was not evaluated. Data on molecular markers and gene alterations were also not included in the SEER database, so that the influence of these factors could not be evaluated. Despite these limitations, the strengths of current study include a large sample size which facilitated multivariable adjustment and continuous analysis of age effects on dependent variables.

Despite a dramatic rise in incidence, mortality due to thyroid cancer has increased only slightly over the years (roughly 2% over 25 years) [31]. In recent years, physicians have increasingly begun to apply active surveillance as a valid approach to manage low-risk cancers [32, 33]. The 2015 ATA guidelines adopted active surveillance as a management option for low-risk PTMC, as an alternative to thyroidectomy [34]. Microscopic lymph node positivity conveys a much smaller risk of recurrence than do macroscopic clinically apparent loco-regional metastases [35].

## Conclusion

Our study demonstrated that LNM may disappear spontaneously and tumors may become smaller in some young and middle-aged PTC patients, and from this perspective, our results imply that low-risk PTC patients may be managed with active surveillance.

## Supplementary information

**Additional file 1: Figure S1.** The distribution of age at diagnosis in thyroid cancer patients. **A**, Patients with papillary thyroid carcinoma (PTC); **B**, Patients with follicular thyroid carcinoma (FTC); **C**, Patients with PTC classified by lymph node status. The median age was 45 years (range, 18–99 years) in PTC and 50 (18–94) in FTC. **Abbreviations**: LNM, lymph node metastasis.

**Additional file 2: Table 1.** Correlation Analysis of Age at diagnosis and Lymph Node Metastasis Rate in Patients with PTC, Aged 18 to 59.

## Data Availability

We received permission to access the research data file in the SEER program from the National Cancer Institute, United States (reference number 15218-Nov2017).

## Abbreviations

AJCC: American joint committee on cancer
ATA: American thyroid association
DTC: Differentiated thyroid carcinoma
ETE: Extrathyroidal extension
FTC: Follicular thyroid carcinoma
LNM: Lymph node metastasis
OR: Odds ratio
OS: Overall survival
PTC: Papillary thyroid carcinoma
PTMC: Papillary thyroid microcarcinoma
SEER: Surveillance, epidemiology, and end results
