# Supplementary figures and images for: Lymph node metastasis in young and middle-aged papillary thyroid carcinoma patients: a SEER-based cohort study
Source: BMC Cancer. 2020 Mar 4;20:181. doi: 10.1186/s12885-020-6675-0 (PMC7057480; doi:10.1186/s12885-020-6675-0)

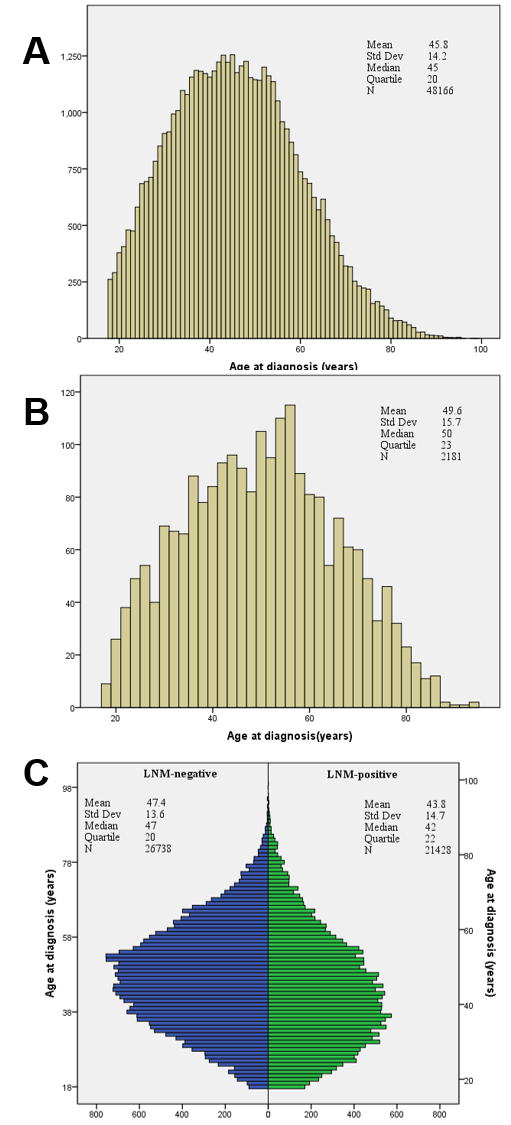

Supplement: Supplementary file 1 — Additional file 1: Figure S1. The distribution of age at diagnosis in thyroid cancer patients. A, Patients with papillary thyroid carcinoma (PTC); B, Patients with follicular thyroid carcinoma (FTC); C, Patients with PTC classified by lymph node status. The median age was 45 years (range, 18–99 years) in PTC and 50 (18–94) in FTC. Abbreviations: LNM, lymph node metastasis. [file 12885_2020_6675_MOESM1_ESM.tiff]
