# Supplementary material for: Lymph node metastasis in young and middle-aged papillary thyroid carcinoma patients: a SEER-based cohort study
Source: BMC Cancer. 2020 Mar 4;20:181. doi: 10.1186/s12885-020-6675-0 (PMC7057480; doi:10.1186/s12885-020-6675-0)
Supplement: Supplementary file 2 — Additional file 2: Table 1. Correlation Analysis of Age at diagnosis and Lymph Node Metastasis Rate in Patients with PTC, Aged 18 to 59. [file 12885_2020_6675_MOESM2_ESM.doc]

| **Appendix** **Table 1.** Correlation Analysis of Age at diagnosis and Lymph Node Metastasis Rate in Patients with PTC, Aged 18 to 59 | | | | |
| --- | --- | --- | --- | --- |
| **Patients** | **Slope** | **Y-intercept** | **R2** | **P value** |
| **Total PTC** | -0.75 | 74.46 | 0.932 | <.0001 |
| **Total PTMC** | -0.54 | 51.61 | 0.755 | <.0001 |
| **T0 and T1a PTC** | -0.53 | 48.78 | 0.764 | <.0001 |
| **T1b PTC** | -0.68 | 73.57 | 0.874 | <.0001 |
| **T2 PTC** | -0.70 | 80.34 | 0.834 | <.0001 |
| **T3 PTC** | -0.71 | 94.10 | 0.880 | <.0001 |
| **T4 PTC** | -0.35 | 100.40 | 0.342 | <.0001 |
| **Male PTC** | -0.62 | 87.68 | 0.787 | <.0001 |
| **Female PTC** | -0.86 | 77.23 | 0.951 | <.0001 |

**Abbreviations**: PTC, papillary thyroid carcinoma; PTMC, papillary thyroid microcarcinoma.
